# Satisfaction in population-based cancer screening in a Chinese rural high-risk population: the Yangzhong early diagnosis and treatment of upper gastrointestinal cancer

**DOI:** 10.1186/s12913-022-08076-1

**Published:** 2022-05-19

**Authors:** Xiang Feng, Jinhua Zhu, Zhaolai Hua, Qin Zhou, Aiwu Shi, Tongqiu Song, Shenghua Yao, Ru Chen, Wenqiang Wei, Jinyi Zhou

**Affiliations:** 1 Institute of Tumour Prevention and Control, People’s Hospital of Yangzhong City, Yangzhong, China; 2 Endoscopy Center, People’s Hospital of Yangzhong City, Yangzhong, China; 3 Cancer Registry Office, National Cancer Center/National Clinical Research Center for Cancer/Cancer Hospital, Chinese Academy of Medical Science and Peking Union Medical College, Beijing, China; 4 Department of Non-Communicable Disease Prevention, Jiangsu Provincial Center for Disease Control and Prevention, Nanjing, China

## Abstract

**Background:**

Screening for upper gastrointestinal cancer (UGC) effectively reduces morbidity and mortality in gastric and esophageal cancers. It is considered one of the effective measures for cancer control in China, but studies on its functional quality are lacking. Our study assessed the quality of screening service funded by Upper Gastrointestinal Cancer Early diagnosis and treatment (UGCEDAT) and its correlation in Yangzhong People’s hospital, China.

**Methods:**

A cross-sectional study was conducted among 516 screening users at a screening centre in Yanghzong People’s hospital from April to July 2021. The service quality questionnaire (SERVQUAL) based on the service quality gap (SQG) model was adopted. We calculated the mean scores of perceptions and expectations and their gap. To determine the association between overall SQG and related features of participants, we used a multivariate logistic regression.

**Results:**

The average scores of screening service users’ perceptions and expectations were 4.05 and 4.55, respectively. The SQG of five dimensions (tangibles, reliability, responsiveness, assurance and empathy) were negative, and the overall SQG was -0.51. The responsiveness dimension had the largest gap, and tangibles had the smallest gap. Occupation status (AOR: 0.57; CI: 0.37–0.89), health self-assessment (AOR: 4.97; CI: 1.35–18.23), endoscopy experience (AOR: 0.55; CI: 0.38–0.81), distance from screening hospital (AOR: 1.85; CI: 1.25–2.73) and frequency of visit (AOR: 1.65; CI: 1.10–2.46) were associated with the overall SQG.

**Conclusions:**

We observed a negative gap between perceptions and expectations of the function quality of screening service, implying a high dissatisfaction across different dimensions. Service providers should take adequate measures to bridge the dimension with the largest quality gap. Meanwhile, attention should be paid to identifying the influencing factors of the overall SQG and the characteristics of dimensional expectations and perceptions to improve the effectiveness of the screening program.

**Supplementary Information:**

The online version contains supplementary material available at 10.1186/s12913-022-08076-1.

## Background

Globally, cancer of the upper gastrointestinal, represented by gastric and esophageal cancers, is a common malignancy that seriously threatens the health and property of the population [1, 2]. Numerous cancer epidemiological studies have shown that the upper gastrointestinal cancer (UGC) is becoming more pronounced globally and has become one of the major components of cancer-related morbidity and mortality in the population [2, 3]. In response to the severe trend of the UGC epidemic, some countries with a high incidence (Japan, Korea, China, etc.) attach great importance to the vital role of screening in cancer prevention and treatment. They have launched the National Cancer Screening Program (NCSP), which aims to detect suspicious or early lesions through regular upper gastrointestinal endoscopic screening in high-risk or average-risk population groups for targeted intervention and treatment. Furthermore, staff also provide various health education and promotion related to UGC during the screening process [4–9]. Screening practice has shown that NCSP can identify many patients with precancerous disease and lesions, reduce the number of advanced cancers, or improve their prognosis. One-time endoscopic screening programs have been reported to reduce UGC incidence by 23%-43% and mortality by 53%-57%, respectively, in high-risk areas [10, 11]. Meanwhile, several studies showed that screening for UGC is cost-effective [12, 13]. As a result, population-based screening of UGC in China has gradually begun to shift towards opportunistic screening to expand the coverage and speed of the screening.

To maximize the benefits of cancer screening, the targeted population must participate in it as much as possible, which places high demands on population adherence. However, the participation rate of the targeted population in the UGC screening programme in Henan province, China, has been reported to be only 18.4% [14]. A project summary of UGC screening reported that the quality of screening services in China was not optimistic, characterized by low early diagnosis rates, low 5-year survival rates, weak service levels and poor population adherence, especially in rural areas [15]. More Chinese scholars have studied the technical quality of screening services for UGC (early diagnosis rates, screening results and 5-year survival rates, etc.). Few studies have focused on the functional quality (service levels, communication skills, screening waiting times, etc.). Although technical quality is essential for screening, it is assessed chiefly as an afterthought from the service provider's perspective and lacks service users' perceptions. The assessment of functional quality can fill the gap because it relates to service users' access to healthcare services. As service recipients, screened participants can judge based on their perceptions [16]. It is reflected in satisfaction, which includes ratings of the screening environment (e.g. equipment, the comfort of the waiting area), accessibility and convenience, physician competence (e.g. attitude, communication and explanation), level of medical skill and discomfort (e.g. experiencing pain or fear) [7, 17, 18].

With the change in the medical paradigm and the improvement in the population's standard of living, satisfaction has become one of the critical outcome indicators of healthcare services. It is recommended by the WHO [19, 20]. Understanding satisfaction with screening can, on the one hand, make us aware of shortcomings in the design of the screening programme, process arrangements and service levels. On the other hand, improved service quality can help ameliorate compliance among the targeted population and thus expand the health benefits of screening.

The objectives of this study were to first investigate expectations before screening and actual perceived quality after screening among participants attending UGC screening in Yangzhong City, China, using the service quality questionnaire (SERVQUAL) instrument based on the service quality gap (SQG) model, and then to calculate the gap between expectations and perceptions to determine satisfaction levels and undesirable quality dimensions. Our study also aimed to explore the factors associated with the overall SQG.

## Methods

### Study design and setting

This cross-sectional study was conducted from 9 April to 5 July 2021 in the People's hospital of Yangzhong City, Jiangsu province, a typical high-risk area for UGC in the southeast region of China.

The hospital has been undertaking the Upper Gastrointestinal Cancer Early Diagnosis and Treatment (UGCEDAT) since 2006, screening 2,000 high-risk cases from rural areas each year while participating in an esophageal cancer-specific cohort project in 2016 [9]. In brief, the project adopted a cluster sampling method in which local permanent residents (male and female) aged 40–69 years are considered at high risk of UGC. Recruitment is carried out village by village. While screening and early diagnosis benefits are promoted, village doctors and local staff will notify all target groups to attend the designated hospital for endoscopy. Those willing to do arrive at the hospital and complete the process of informed consent, screening registration, physical examination, epidemiological investigation, laboratory biochemical index testing and endoscopy, respectively, before concluding the screening. In addition, the endoscopic biopsy pathology report will be issued within the next 1–2 weeks and will be distributed by local staff. Specific information can be found in the relevant literature [9, 21].

Therefore, this hospital has abundant experience in UGC screening and is a leader in Jiangsu province. Our survey on screening satisfaction is beneficial to our development and has a significant demonstration and guiding role in optimizing the service among other project sites in the province.

We conducted this study as part of the UGCEDAT. All valid screening subjects (both women and men) as potential respondents unless they met the following exclusion criteria: (1) excluded before screening because of contraindications to endoscopy; (2) were unwilling to participate in the satisfaction survey or refused to sign the informed consent form. The inclusion criteria included: (1) 40–69 years of age; (2) permanent residents in Yangzhong City; and (3) being able to understand the survey procedures and communicate fluently [9, 22].

### Instrument

The SERVQUAL scale based on the SQG model was adopted in our study, a reliable and valid instrument in many real-world settings. The SQG model is based on the definition of service quality as the comparison between what is provided and what is expected and the establishment and understanding of the gaps in the service delivery process [19, 20].Parasuraman, Berry and Zeithaml initially proposed the SQG model to assess the perception and decisions of service quality among customers. The original model included ten dimensions. Subsequently, they developed the SERVQUAL [23, 24]. Further, they also developed a modified SERVQUAL scale by reducing the number of dimensions to five (tangibles, reliability, responsiveness, assurance and empathy), containing 22 sub-items for both expectation and perception sections (see Supplementary Table 1) [25].

Ultimately, SERVQUAL has become an effective tool, whose original language was English, for investigating and analyzing the functional quality of service rather than technical ones. Although the tool may have some shortcomings, many scholars still use it when evaluating the quality of services [26]. The tool has been used in a wide range of service sectors, including banking, travel and education, with the health services sector being no exception [27]. Therefore, this tool was chosen by our group.

Since this questionnaire was not applied to the background of China for the first time, the original SERVQUAL was further modified with reference to the improved experience of Fan and Lu et al. [27, 28] and the characteristics of UGC screening so that the SERVQUAL from a different culture can achieve cultural equivalence among different populations and enable the survey respondents to understand the questionnaire items better. Firstly, the standard SERVQUAL was translated into Chinese by two of the authors (XF and TQS). Then the translated version was given to the experts involved in our study for discussion and revision until the experts' opinions on its ambiguities were unified. Finally, the revised questionnaire was translated back into English by a PhD in epidemiology from the Chinese Academy of Sciences (RC) to ensure the accuracy of the translation. The final revised version of the SERVQUAL contains 22 items from the five dimensions, both in the expectation and perception sections. Each dimension has 1- 5 items and was scored by a 5-point Likert scale (from strongly agree to strongly disagree, see Supplementary Table 2).

The reliability and consistency of the questionnaire were confirmed by Cronbach’s Alpha (α = 0.888 for the expectation section, α = 0.671 for the perception section). Kaiser–Meyer–Olkin (KMO) and Bartlett’s test was performed to confirm the validity of our questionnaire (KMO = 0.906 > 0.60, chi-square value = 4666.434, *p* < 0.001 for expectation section; KMO = 0.752 > 0.60, chis-square value = 1860.121, *p* < 0.001 for perception section). Confirmatory factor analysis indicated that the extracted factors contributed to 60.32% and 55.31% of the variance for expectations and perceptions using the Varimax method, respectively.

In addition, basic information (gender, age, marital status, education, occupation status, residence, average annual family income), health-related characteristics( health self-assessment, screening anxiety, endoscopy experience, distance from screening hospital, screening purpose, frequency of visit, common chronic diseases, and screening results) were included in the questionnaire. Screening discomfort and satisfaction were also collected from the respondents (see Supplementary Table 3).

The study’s data collection steps were as follows: (1) After completing screening registration, respondents were invited to independently complete the questionnaire’s baseline and health-related characteristics and expectation section in the epidemiological investigation rooms. (2) After receiving all UGC screening services, they were again invited to complete the remainder of the questionnaire independently in the same places. (3) The research team was responsible for on-site quality control and conducted supplementary surveys for questionnaires with missing values.

### Statistical analysis

All respondents’ data were described with frequency and percentage or mean and standard deviation as appropriate. The SQG of each item was obtained by subtracting expectation (mean) from the perception (mean). SQG for each dimension was calculated by the perceptual mean minus the expected mean of items that composed them. Briefly, the SQG = Perception (P)-Expectation (E). If: E > P (negative gap, dissatisfied). If: E ≤ P (positive gap, satisfied). Paired samples *t*-test was applied to compare differences in expectations and perceptions and identify the dimension and item with the largest gap. One-way logistic regression was used to find the potential factors associated with the overall SQG (*p* < 0.3). Multivariate logistic regression analysis was applied to evaluate independent factors that affect the overall SQG. SPSS 17.0 was the analysis software, and the *p*-value < 0.05 was significant.

## Results

### Basic information of the study population

A total of 627 cases were screened, and 518 satisfaction questionnaires were returned, of which 516 were valid, with a response rate of 82.62% and an effective rate of 99.61%. The non-respondents had mostly similar characteristics to those in the analyses (see Supplementary Table 4). Table 1 shows that among the 516 respondents, the age ranged from 40 to 69 with an average of 56.65 (SD = 7.18) years, and the majority were between 50 to 59 years old (*n* = 257, 49.8%). The participants of the survey were more commonly female (*n* = 311, 60.3%), currently married (*n* = 482, 93.4%), junior high school (*n* = 252, 48.8%), employed (*n* = 398, 77.1%), rural residents (*n* = 424, 82.2%), and their most family income was 30,000–69,999 CNY/year. Meanwhile, Of the participants surveyed, 57.9% reported they were in better health status; 40.1% had screening anxiety; 52.7% had endoscopy experience; 50.4% reported that the time they needed to reach the screening centre was between 30–60 min; 89.5% participated this screening for the purpose of medical examination; 66.9% visited the screening hospital for the first time; 35.3% had common chronic diseases; the screening results among them were mostly normal (90.9%), and 32.8% reported that they had discomfort during the screening.

**Table 1:** Basic information among respondents

Variables	Total (%), *N* = 516	Male (%), *N* = 205	Female (%), *N* = 311
Age in years ^a^	56.62 (7.18)	56.67 (7.40)	56.59 (7.04)
Age			
40–49	86 (16.7)	37 (43.0)	49 (57.0)
50–59	257 (49.8)	98 (38.1)	159 (61.9)
60–69	173 (33.5)	70 (40.5)	103 (59.5)
Marital Status			
Currently married	482 (93.4)	195 (40.5)	287 (59.5)
Others ^b^	34 (6.6)	10 (29.4)	24 (70.6)
Education level			
Primary school and below	189 (36.6)	46 (24.3)	143 (75.7)
Junior high school	252 (48.8)	111 (44.0)	141 (56.0)
Senior high school and above	75 (14.5)	48 (64.0)	27 (36.0)
Occupation Status			
Unemployed ^c^	118 (22.9)	17 (14.4)	101 (85.6)
Employed ^d^	398 (77.1)	188 (47.2)	210 (52.8)
Residence			
Rural	424 (82.2)	176 (41.5)	248 (58.5)
Urban	92 (17.8)	29 (31.5)	63 (68.5)
Family income (CNY/year)			
< 30,000	64 (12.4)	18 (28.1)	46 (71.9)
30,000–69,999	175 (33.9)	79 (45.1)	96 (54.9)
70,000–109,999	135 (26.2)	53 (39.3)	82 (60.7)
≥ 110,000	142 (27.5)	55 (38.7)	87 (61.3)
Health self-assessment			
Very good	113 (21.9)	48 (42.5)	65 (57.5)
Better	299 (57.9)	131 (43.8)	168 (56.2)
General	91 (17.6)	22 (24.2)	69 (75.8)
Poor	13 (2.5)	4 (30.8)	9 (69.2)
Screening anxiety			
Yes	207 (40.1)	51 (24.6)	156 (75.4)
NO	309 (59.9)	154 (49.8)	155 (50.2)
Endoscopy experience			
Yes	272 (52.7)	94 (34.6)	178 (65.4)
NO	244 (47.3)	111 (45.5)	133 (54.5)
Distance from screening hospital			
Less than 30 min	196 (38.0)	83 (42.3)	113 (57.7)
30–60 min	260 (50.4)	102 (39.2)	158 (60.8)
More than 1 h	60 (11.6)	20 (33.3)	40 (66.7)
Screening purpose			
Medical examination	462 (89.5)	179 (38.7)	283 (61.3)
Disease review	54 (10.5)	26 (48.1)	28 (51.9)
The first time of visit			
Yes	345 (66.9)	131 (38.0)	214 (62.0)
NO	171 (33.1)	74 (43.3)	97 (56.7)
Common chronic diseases ^e^			
Yes	182 (35.3)	81 (44.5)	101 (55.5)
NO	334 (64.7)	124 (37.1)	210 (62.9)
Screening results			
Normal (including inflammation)	469 (90.9)	175 (37.3)	294 (62.7)
Precancerous diseases and above ^f^	47 (9.1)	30 (63.8)	17 (36.2)
Screening discomfort ^a g^	4.79 (0.39)	4.82 (0.35)	4.76 (0.41)
< 4.79 (Yes)	169 (32.8)	58 (34.3)	111 (65.7)
≥ 4.79 (No)	347 (67.2)	147 (42.4)	200 (57.6)

^a^ Data summarized as mean (standard deviation)

^b^ Others include unmarried, divorced, widowed, and separated

^c^ Unemployed includes household work, leaving/retired and unemployed/layoffs

^d^ Employed includes agricultural, forestry, livestock and fishery workers, workers, administrative and managerial staff, professional and technical staff (doctors, teachers, scientists), sales and service workers, private owners and others or those who cannot be easily classified

^e^ Common chronic diseases include self-reported of hypertension, diabetes or hyperlipidemia

^f^ Precancerous diseases and above include atrophic gastritis, severe intestinal metaplasia, low-grade neoplasia, high-grade neoplasia and cancer

^g^ The score of each item for screening discomfort ranges from 5 (strongly satisfied) to 1 (strongly dissatisfied) and the overall score was averaged score for this domain; *N*, Frequency

### Perceptions and expectations for five dimensions of screening service

As exhibited in Table 2, the expectation scores were higher than the perceptions scores in all SERVQUAL dimensions, with a significant difference (*p* < 0.001). The overall scores of the perception section were 4.05 ± 0.20, and the empathy dimension had the lowest scores (3.76 ± 0.34). The overall scores of the expectation section were 4.55 ± 0.28, and the tangibles had the lowest scores (4.16 ± 0.60). The most critical dimension was responsiveness, which had the largest SQG. Moreover, the tangibles dimension had the smallest SQG.

**Table 2:** Scores and gaps in the expectation and perception of service quality for five dimensions based on the SERVQUAL scale

SERVQUAL dimensions	Perception	Expectation	SQG ^a^	*t*-value	*p-*value	Rank
Tangibles	3.86 ± 0.31	4.16 ± 0.60	-0.30 ± 0.66	-10.53	0.000	5
Reliability	4.30 ± 0.34	4.83 ± 0.26	-0.53 ± 0.42	-28.44	0.000	4
Responsiveness	4.09 ± 0.30	4.69 ± 0.32	-0.59 ± 0.40	-33.93	0.000	1
Assurance	4.24 ± 0.32	4.79 ± 0.24	-0.56 ± 0.38	-33.45	0.000	2
Empathy	3.76 ± 0.34	4.30 ± 0.35	-0.54 ± 0.45	-27.15	0.000	3
Total	4.05 ± 0.20	4.55 ± 0.28	-0.51 ± 0.34	-33.72	0.000	一

^a^ *SQG* service quality gap

### Perceptions and expectations for each item based on SERVQUAL dimensions

Table 3 compares the perception and expectation scores of each item on the screening service. The expectation scores significantly exceeded perception ones in all items except for items 1, 3 and 20 (*p* < 0.001). The largest gap among them in five dimensions was item 4 for tangibles, 5 for reliability, 10 for responsiveness, 15 for assurance, and 19 for empathy. The participants had the highest perceptions for item 9 (4.76 ± 0.44), followed by 16 (4.62 ± 0.49) and 21 (4.58 ± 0.50). The lowest perception was item 4 (3.18 ± 0.61). The highest expectations of them was item 9 (4.99 ± 0.08), followed by 21 (4.94 ± 0.24) and 17 (4.92 ± 0.28). There was a lowest expectation in terms of 22 (3.91 ± 0.57).

**Table 3:** Scores and gaps in the expectation and perception of service quality for each item among the five dimensions

Perception		Expectation		SQG ^a^	Rank	*t-*value	*p*-value
Dimension/Item	Mean ± SD	Dimension/Item	Mean ± SD				
Tangibles		Tangibles					
P1	4.00 ± 0.53	E1	4.08 ± 0.77	-0.08 ± 0.95	3	1.95	0.052
P2	4.07 ± 0.43	E2	4.19 ± 0.57	-0.12 ± 0.73	2	3.88	0.000
P3	4.17 ± 0.42	E3	4.22 ± 0.56	-0.05 ± 0.71	4	1.50	0.135
P4	3.18 ± 0.61	E4	4.15 ± 0.95	-0.97 ± 1.08	1	20.28	0.000
Reliability		Reliability					
P5	4.01 ± 0.57	E5	4.76 ± 0.43	-0.75 ± 0.66	1	25.75	0.000
P6	4.38 ± 0.55	E6	4.75 ± 0.43	-0.38 ± 0.71	4	12.04	0.000
P7	4.22 ± 0.61	E7	4.80 ± 0.40	-0.58 ± 0.70	3	18.82	0.000
P8	4.14 ± 0.69	E8	4.83 ± 0.38	-0.69 ± 0.73	2	21.47	0.000
P9	4.76 ± 0.44	E9	4.99 ± 0.08	-0.23 ± 0.43	5	12.23	0.000
Responsiveness		Responsiveness					
P10	3.93 ± 0.58	E10	4.77 ± 0.42	-0.84 ± 0.67	1	28.56	0.000
P11	4.47 ± 0.52	E11	4.91 ± 0.29	-0.44 ± 0.51	4	19.50	0.000
P12	4.03 ± 0.42	E12	4.56 ± 0.58	-0.52 ± 0.61	3	19.64	0.000
P13	3.92 ± 0.57	E13	4.50 ± 0.52	-0.58 ± 0.66	2	19.96	0.000
Assurance		Assurance					
P14	3.99 ± 0.46	E14	4.51 ± 0.54	-0.52 ± 0.72	3	16.45	0.000
P15	4.03 ± 0.64	E15	4.83 ± 0.39	-0.80 ± 0.64	1	28.44	0.000
P16	4.62 ± 0.49	E16	4.92 ± 0.27	-0.30 ± 0.47	4	14.28	0.000
P17	4.31 ± 0.52	E17	4.92 ± 0.28	-0.60 ± 0.53	2	25.85	0.000
Empathy		Empathy					
P18	3.41 ± 0.63	E18	4.10 ± 0.60	-0.69 ± 0.75	2	21.02	0.000
P19	3.49 ± 0.91	E19	4.58 ± 0.50	-1.08 ± 1.06	1	23.24	0.000
P20	3.93 ± 0.59	E20	3.97 ± 0.68	-0.03 ± 0.86	5	0.87	0.383
P21	4.58 ± 0.50	E21	4.94 ± 0.24	-0.36 ± 0.50	4	16.49	0.000
P22	3.39 ± 0.72	E22	3.91 ± 0.57	-0.51 ± 0.88	3	13.29	0.000

^a^ *SQG *service quality gap, *SD *standard deviation

### Factors associated with the overall service quality gap in screening service

We first converted the total SQG score into a binary variable with the mean (-0.51) as the cutoff point (0 =  < -0.51, 1 =  ≥ -0.51). Table 4 summarizes the factors associated with the overall SQG (0 = worse SQG, 1 = better SQG) based on multivariate logistic regression analysis. Participants in employed status had 0.5-folds lower odds of higher service quality (satisfaction) compared to those who had no job (OR = 0.57, 95%CI = 0.37–0.89). Compared with participants with poor health status, participants with general health status were a greater possibility of higher service quality (OR = 4.97, 95%CI = 1.35–18.23). Participants with no endoscopy experience were less likely to give higher service quality evaluation (OR = 0.55, 95%CI = 0.38–0.81). Meanwhile, respondents who reported that the distance from the screening hospital was between 30 to 60 min and it was not the first time to visit our screening hospital had 1.85 (OR = 1.85, 95%CI = 1.25–2.73) and 1.65 (OR = 1.65, 95%CI = 1.10–2.46) times higher odds of higher service quality compared to their counterparts.

**Table 4:** Multivariate logistic regression analysis for factors associated with a better service quality gap (≥ -0.51)

Variables	OR	95%CI	*p*-value
Model 1			
Gender (Male)	1.13	0.79–1.60	0.508
Female			
Age (40–49)			0.349
50–59	1.27	0.78–2.08	0.335
60–69	1.47	0.87–2.47	0.148
Marital Status (Others)			
Currently married	0.64	0.31–1.31	0.221
Education level (Primary school and below)			0.110
Junior high school	0.78	0.53–1.14	0.196
Senior high school and above	0.57	0.33–0.98	0.042
Occupation status (Unemployed)			
Employed	0.58	0.38–0.88	0.011
Residence (Rural)			
Urban	0.88	0.56–1.38	0.577
Family income (< 30,000 CNY/year)			0.104
30,000–69,999	1.15	0.65–2.04	0.635
70,000–109,999	1.01	0.56–1.83	0.978
≥ 110,000	0.66	0.37–1.20	0.177
Health self-assessment (Poor)			0.013
Very good	1.79	0.52–6.14	0.357
Better	2.36	0.71–7.82	0.161
General	4.15	1.18–14.54	0.026
Screening anxiety (Yes)			
NO	0.99	0.70–1.41	0.958
Endoscopy experience (Yes)			
NO	0.63	0.44–0.89	0.009
Distance from screening hospital (Less than 30 min)			0.039
30–60 min	1.61	1.10–2.33	0.013
More than 1 h	1.53	0.86–2.74	0.151
Screening purpose (Medical examination)			
Disease review	1.20	0.68–2.11	0.534
The first time of visit (Yes)			
NO	1.32	0.91–1.90	0.142
Common chronic diseases (Yes)			
NO	1.22	0.85–1.75	0.283
Screening results (Normal)			
Precancerous disease and above	0.61	0.33–1.12	0.112
Screening discomfort (Yes)			
No	0.84	0.58–1.22	0.360
Model 2 ^*^			
Occupation status (Unemployed)			
Employed	0.57	0.37–0.89	0.013
Health self-assessment (Poor)			0.010
Very good	2.09	0.58–7.56	0.261
Better	2.98	0.85–10.41	0.087
General	4.97	1.35–18.23	0.016
Endoscopy experience (Yes)			
NO	0.55	0.38–0.81	0.002
Distance from screening hospital (Less than 30 min)			0.009
30–60 min	1.85	1.25–2.73	0.002
More than 1 h	1.48	0.81–2.70	0.203
The first time of visit (Yes)			
NO	1.65	1.10–2.46	0.015

^*^ Model 2 included variables found to be significant in Model 1 with a *p-*value < 0.3

## Discussion

The study was designed to evaluate the service gap and its influencing factors of provided UGC screening in the People’s Hospital of Yangzhong City in southeast China using the SERVQUAL scale and help managers develop appropriate promotion strategies.

Generally, the SQG for all dimensions of the screening service were negative, and the overall SQG was -0.51, implying that service users' expectations were higher than their perceptions and they were dissatisfied with all five dimensions, consistent with the results of other studies [29, 30]. The SQG in the provided service in health centers in Mashhad of Iran was -1.707 [29]. Evaluation of outpatient service quality in a hospital in the Eastern Province of Saudi Arabia showed that the SQG was -1.200 [30]. Aghamolaei and Eftekhaari revealed that the SQG in the provided service was -1.29 [31]. In contrast, some Chinese scholars found relatively small SQG. A study aimed to determine the SQG of provided service among twenty-seven hospitals in 15 provinces found that the SQG was -0.3888 [27] and the SQG found in the provided nursing service via SERVQUAL scale in a tertiary hospital of Hubei Province, China, was -0.35 [32]. The overall SQG presented in different studies are diverse due to differences in culture, economy, health policy, the type of health services and the targeted population included in studies. The overall SQG in our study is at a medium level compared to the literature mentioned above [27, 29–32]. However, the prevalent SQG suggests a deficiency in screening services and reminds managers to take early steps to close the gap.

The present study revealed that service users' expectations were high, and the overall expectations were 4.55. The expectations were ranked from high to low: reliability, assurance, responsiveness, empathy and tangibles. Reliability, assurance, and responsiveness were the top three dimensions with expectation scores of more than 4.50, consistent with one previous study performed by Lu et al. [28]. In that study, the highest three expectation scores were reliability (4.73), assurance (4.66), and responsiveness (4.60), respectively. This finding was consistent with the philosophy of health care displayed by Chinese residents today. During the consultation process, patients are very concerned about the professionalism, timeliness of the medical services, and attitude of the medical staff [33, 34]. Meanwhile, we found that the expectations of tangibles and empathy were at a relatively low level. This indicated that the needs of service users are still at the basic stage, and the need for higher dimensions such as tangibles and empathy has not yet been fully released, according to Maslow's hierarchy of needs [27]. Screening service providers should improve tangibles and empathy dimensions because this is necessary to improve overall service quality and in line with person-centred health care.

Our study revealed that the top three dimensions with perceptions were reliability, assurance, and responsiveness, with mean scores of more than 4.00, which indicated that screening service users have a good perception of quality in these three dimensions. Specifically, our study's highest perception score was related to the reliability dimension (4.30), consistent with several previous studies [28, 35]. However, Fan et al. and Aghamolaei et al. mentioned the highest perceptions in the assurance dimension [27, 31]. Sharifi et al. showed that tangibles was the highest-performing dimension [29], and some other studies revealed that empathy was their highest-performing dimension [30, 36]. In conclusion, different studies tend to present different findings, which may be attributed to the heterogeneity of the types of health services, and the capacity of the services. We also found that tangibles (3.86) and empathy (3.76) perception scores were less than 4. Specifically, item 4 ("Materials associated with the service are visually appealing.") in the tangibles dimension and item 22 ("Workers at the Screening Centre understand the special needs of the patient and the family.") in the empathy dimension were the lowest-performing items among 22 items, which pointed out the direction of quality improvement.

As mentioned above, the SQG among the five dimensions were all negative, and their rank was as follows (high to low): responsiveness, assurance, empathy, reliability and tangibles. In other words, the largest SQG was in the responsiveness dimension. This dimension includes the willingness to help patients and families and provide prompt service [37]. The largest gaps in responsiveness were items 10 ("Workers at the Screening Centre tell you exactly when the care will be performed.") and 13 ("Workers at the Screening Centre are never too busy to respond to the patient's or your requests"). This could be related to complex screening procedures and insufficient service awareness among staff. Specifically, screening for UGC involves some necessary procedures such as screening registration, ECG, epidemiological investigations, endoscopy and biochemical index tests [9, 11, 22]. These processes take varying times, making it impossible to determine precise service times. In addition, the lack of service awareness among staff may overlook the needs of service users.

The SQG for assurance and empathy dimensions were ranked second and third, respectively. Assurance refers to the level of competence, courtesy, credibility and security [37]. The greatest gaps in this dimension were items 15 ("You feel safe for the patient's care by the Screening Centre"). and 17 ("Workers at the Screening Centre have the knowledge to answer your questions."). Lack of experience with endoscopy or unfamiliarity with the investigation process may lead to service users feeling unsafe, with 47.3% of the subjects in this survey having never experienced endoscopy before. Hence, there is a need to enhance health education before screening to increase service users' knowledge of endoscopy and the service process to reduce their feelings of unease. In addition, the large SQG of item 17 may be due to the high expectations of service users because the item's perception score is as high as 4.31. Empathy is a dimension worth focusing on. With improved living standards, service users will pay more and more attention to this dimension [27]. Item 19 ("The Screening Centre has operating hours convenient to its patients and families.") in the empathy dimension had the largest SQG among all items. The working arrangements for the screening service may be a reason for this. Because screening needs to be done on an empty stomach considering the characteristics of endoscopy, it is usually scheduled in the morning, so it is impossible to set a flexible screening time to meet the diverse needs of the targeted population. Although it is difficult to adjust the opening hours of screening, we can improve the perception of this dimension by providing personalized services, actively caring about the discomfort and bad emotions of the screened participants and solving them promptly. Under the premise of fixed expectations, improvement measures can effectively improve the gap in empathy because this dimension currently has the lowest perception score.

The SQG for the reliability and tangibles dimensions were ranked fourth and fifth, respectively. The greatest gaps in the reliability dimension were items 5 ("When the Screening Centre promises to do something by a certain time, it does so.") and 8 ("The Screening Centre provides its service at the time it promises to do so."). Combining the expectation and perception scores of the above two items, we can find that they were both in the high expectation and high perception levels. Reducing their SQG can start by reducing excessive expectations and improving actual perception simultaneously. Hence, on the one hand, the screening managers should inform the service users that the entire screening process may take a long time and do an excellent job of explaining to reduce the users' excessive psychological expectations. On the other hand, the staff should be urged to fulfil the promised service, and managers should pass the different assessments to improve efficiency. The tangibles dimension has the smallest SQG, and the greatest gap in this dimension was item 4 ("Materials associated with the service are visually appealing."). This suggested that we should use a diverse range of posters, brochures, pictures and videos to carry out our services to increase the appeal, rather than just relying on large, unattractive paragraphs of text.

Some studies examined the association between demographic characteristics and SQG. Nevertheless, these studies’ findings are conflicting [38]. In the present study, not only did we explore the relationship between SQG and demographic characteristics, but we also incorporated several health-related characteristics and made some new findings by running a multivariate logistic regression model. Our study showed that SQG was associated with occupational status, health self-assessment, endoscopy experience, distance from screening hospital and frequency of visit.

Consistent with previous studies [39, 40], we found that being employed compared to unemployed was associated with lower odds of higher service quality (satisfaction). Meanwhile, participants who reported that the distance from the screening hospital was less than 30 min had lower odds of higher service quality than the distance between 30–60 min. Those can be explained by socioeconomic status. Whether in terms of education level or economic income, working residents and residents with better medical access have relatively good socioeconomic status, especially in Chinese rural areas. As a result, they are more aware of the services they should receive, leading to higher expectations. If the service fails to reach the expected level, they are more likely to express their dissatisfaction [36]. Moreover, the advantages of cultural literacy and resource capabilities enable them to evaluate services more objectively and impartially. If there are indeed deficiencies in the screening services, it is easier to be discovered [36]. However, our findings differ from some local studies, whereby having no jobs was instead associated with lower satisfaction or occupation status was not associated with satisfaction [41, 42].

Furthermore, compared to participants with poor health status, we found that participants with general health status were more likely to have higher service quality, consistent with previous studies [43, 44]. One possible explanation for the difference might be that participants with poor health status usually suffer from acute or chronic conditions. The varying degrees of disease symptoms lead to a lower quality of life, increasing expectations for screening services. However, our screening service is only used for early diagnosis and does not involve treating the diseases, reducing their perceived quality [9, 12, 22]. Participants who had endoscopy experience were more likely to have higher service quality. This is mainly because this screening service is provided by specialized clinics and professional screening personnel [9, 12, 22]. Compared with outpatient endoscopy, the waiting time, service attitude and doctor-patient communication time are better. Participants who have been to our hospital before were less likely to have higher service quality. Different findings were noted in Slovenia and Saudi Arabia [45, 46]. Usually, the regularity of patients to medical institutions enables service users to understand the shortcomings of the service provider, thus reducing unreasonable expectations and increasing satisfaction [47]. The reason for this contradiction may be that compared to outpatient services, screening services are not only complex and time-consuming procedures but also generate a certain amount of discomfort, resulting in poorer service quality scores.

There were some limitations in our study. First, the data in this study are from only one time period and do not reflect the overall performance of screening services. Meanwhile, the research data mainly comes from UGCEDAT in Yangzhong City and cannot be generalized to the whole country. However, our research made a good demonstration for service quality evaluation of cancer screening. Second, because of the nature of the cross-sectional, we can not determine the correlates of SQG in the present study are causal. Third, the translation of SERVQUAL in the Chinese context may affect the study’s findings, despite the quality control. Therefore, our research team’s future directions are developing the service quality evaluation scale for cancer screening and the in-depth study of the screening service quality at multiple time points and centers.

## Conclusions

We concluded that the functional quality of UGC screening services in Yangzhong City has failed to meet participants’ expectations in all dimensions, and it is generally dissatisfied. This suggests that screening providers and policymakers need to adjust service plans and content timely to reduce the quality gap. Reliability had the highest expectations, and empathy had the lowest perceptions. The largest gap was for the responsiveness dimension, which determines this dimension should be focused on firstly. Meanwhile, influencing factors of overall SQG were occupational status, health self-assessment, endoscopy experience, distance from screening hospital and the frequency of visit, respectively. Hence, service providers should pay attention to identifying key influencing factors of SQG and the characteristics of dimensional expectations and perceptions to improve efficiency in quality improvement.

## Supplementary Information

**Additional file 1.**

## Data Availability

Data are available on reasonable request.

## Abbreviations

UGC: Upper gastrointestinal cancer
UGCEDAT: Upper Gastrointestinal Cancer Early diagnosis and treatment
SERVQUAL: Service quality questionnaire
SQG: Service quality gap
