# Supplementary material for: Satisfaction in population-based cancer screening in a Chinese rural high-risk population: the Yangzhong early diagnosis and treatment of upper gastrointestinal cancer
Source: BMC Health Serv Res. 2022 May 19;22:675. doi: 10.1186/s12913-022-08076-1 (PMC9121570; doi:10.1186/s12913-022-08076-1)
Supplement: Supplementary file 1 — Additional file 1. [file 12913_2022_8076_MOESM1_ESM.docx]

| Table 1 Components of the original modified SERVQUAL |
| --- |
| 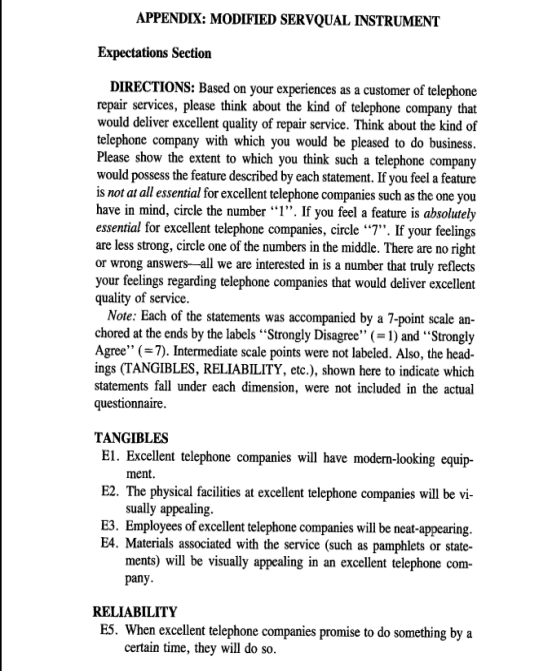 |

| Table 1 Continued |
| --- |
| 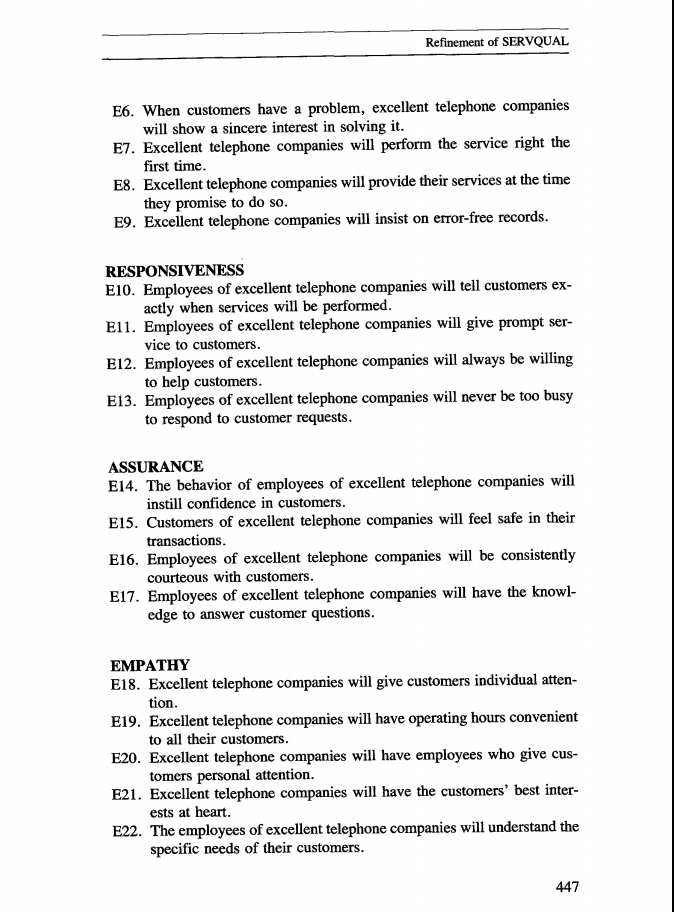 |

| Table 1 Continued |
| --- |
| 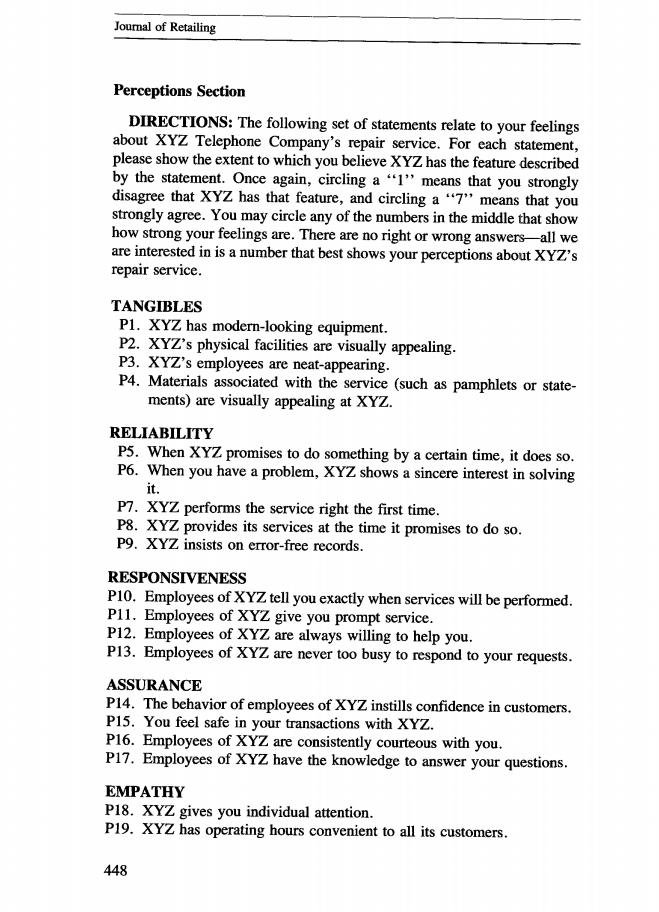 |

| Table 1 Continued |
| --- |
| 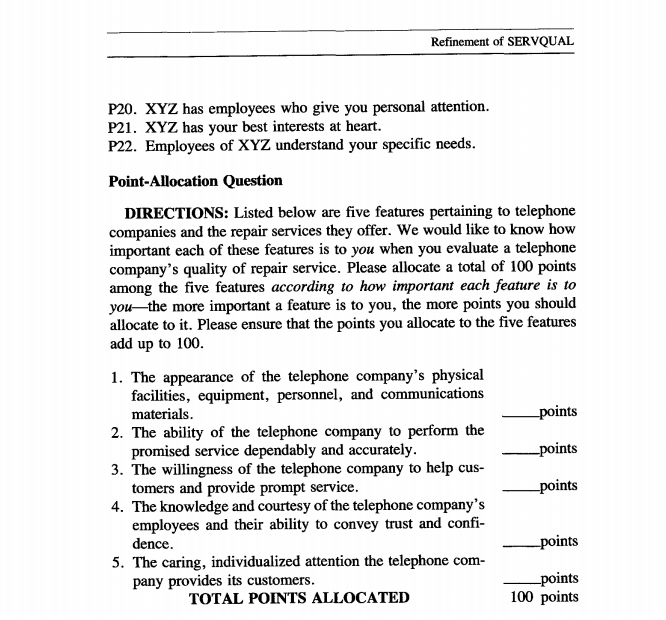 |

| Table 2 Items of the SERVQUAL scale questionnaire used in our study | |
| --- | --- |
| Expectation section | Perception section |
| **Tangibles** (physical facilities, equipment and appearance of personnel) | **Tangibles** (physical facilities, equipment and appearance of personnel) |
| E1. The Screening Centre will have modern-looking equipment. | P1. The Screening Centre has have modern-looking equipment. |
| E2. The Screening Centre‘s physical facilities will be visually appealing. | P2. The Screening Centre‘s physical facilities are visually appealing. |
| E3. The Screening Centre‘s workers will be neat-appearing. | P3. The Screening Centre‘s workers are neat-appearing. |
| E4. Materials associated with the service will be visually appealing. | P4. Materials associated with the service are visually appealing. |
| **Reliability** (ability to perform the promised service dependably and accurately) | **Reliability** (ability to perform the promised service dependably and accurately) |
| E5. When the Screening Centre promises to do something by a certain time, it will do so. | E5. When the Screening Centre promises to do something by a certain time, it does so. |
| E6. When the patients or you have a problem, the Screening Centre will show a sincere interest in solving it. | E6. When the patients or you have a problem, the Screening Centre shows a sincere interest in solving it. |
| E7. The Screening Centre will perform the service right the first time. | E7. The Screening Centre performs the service right the first time. |
| E8. The Screening Centre will provide its service at the time it promises to do so. | E8. The Screening Centre provides its service at the time it promises to do so. |
| E9. The Screening Centre will insist on error-free records. | E9. The Screening Centre insists on error-free records. |
| **Responsiveness** (willingness to help patients and families and provide prompt service) | **Responsiveness** (willingness to help patients and families and provide prompt service) |
| E10. Workers at the Screening Centre will tell you exactly when the care will be performed. | E10. Workers at the Screening Centre tell you exactly when the care will be performed. |
| E11. Workers at the Screening Centre will give the patient prompt care. | E11. Workers at the Screening Centre give the patient prompt care. |
| E12. Workers at the Screening Centre will always be willing to help you and the patient. | E12. Workers at the Screening Centre are always willing to help you and the patient. |
| E13. Workers at the Screening Centre will never be too busy to respond to the patient’s or your requests. | E13. Workers at the Screening Centre are never too busy to respond to the patient’s or your requests. |
| **Assurance** (including competence, courtesy, credibility and security) | **Assurance** (including competence, courtesy, credibility and security) |
| E14. The behavior of the workers at the ICU will instill confidence in the patient and the family. | E14. The behavior of the workers at the ICU instills confidence in the patient and the family. |
| E15. You will feel safe for the patient’s care by the Screening Centre. | E15. You feel safe for the patient’s care by the Screening Centre. |
| E16. Workers at the Screening Centre will be consistently courteous with the patient and the family. | E16. Workers at the Screening Centre are consistently courteous with the patient and the family. |
| E17. Workers at the Screening Centre will have the knowledge to answer your questions. | E17. Workers at the Screening Centre have the knowledge to answer your questions. |
| **Empathy** (caring and individualized attention that the Screening centre provides to its patients and families) | **Empathy** (caring and individualized attention that the Screening centre provides to its patients and families) |
| E18. The Screening Centre will give you and the patient individual attention. | E18. The Screening Centre gives you and the patient individual attention. |
| E19. The Screening Centre will have operating hours convenient to its patients and families. | E19. The Screening Centre has operating hours convenient to its patients and families. |
| E20. The Screening Centre will have workers who give the patient and the family personal attention. | E20. The Screening Centre has workers who give the patient and the family personal attention. |
| E21. The Screening Centre will have the best interest of the patient and the family at heart. | E21. The Screening Centre has the best interest of the patient and the family at heart. |
| E22. Workers at the Screening Centre will understand the special needs of the patient and the family. | E22. Workers at the Screening Centre understand the special needs of the patient and the family. |

Each dimension and item has 1- 5 items and was scored by a 5-point Likert scale (from strongly agree to strongly disagree).

| Table 3 Upper Gastrointestinal Cancer Screening Questionnaire | | |
| --- | --- | --- |
| Measure | Description |  |
| Service Quality | Expectation section | Perception section |
|  | **Tangibles** (physical facilities, equipment and appearance of personnel) | **Tangibles** (physical facilities, equipment and appearance of personnel) |
|  | E1. The Screening Centre will have modern-looking equipment. | P1. The Screening Centre has have modern-looking equipment. |
|  | E2. The Screening Centre‘s physical facilities will be visually appealing. | P2. The Screening Centre‘s physical facilities are visually appealing. |
|  | E3. The Screening Centre‘s workers will be neat-appearing. | P3. The Screening Centre‘s workers are neat-appearing. |
|  | E4. Materials associated with the service will be visually appealing. | P4. Materials associated with the service are visually appealing. |
|  | **Reliability** (ability to perform the promised service dependably and accurately) | **Reliability** (ability to perform the promised service dependably and accurately) |
|  | E5. When the Screening Centre promises to do something by a certain time, it will do so. | P5. When the Screening Centre promises to do something by a certain time, it does so. |
|  | E6. When the patient or you have a problem, the Screening Centre will show a sincere interest in solving it. | P6. When the patient or you have a problem, the Screening Centre shows a sincere interest in solving it. |
|  | E7. The Screening Centre will perform the service right the first time. | P7. The Screening Centre performs the service right the first time. |
|  | E8. The Screening Centre will provide its service at the time it promises to do so. | P8. The Screening Centre provides its service at the time it promises to do so. |
|  | E9. The Screening Centre will insist on error-free records. | P9. The Screening Centre insists on error-free records. |
|  | **Responsiveness** (willingness to help patients and families and provide prompt service) | **Responsiveness** (willingness to help patients and families and provide prompt service) |
|  | E10. Workers at the Screening Centre will tell you exactly when the care will be performed. | P10. Workers at the Screening Centre tell you exactly when the care will be performed. |
|  | E11. Workers at the Screening Centre will give the patient prompt care. | P11. Workers at the Screening Centre give the patient prompt care. |
|  | E12. Workers at the Screening Centre will always be willing to help you and the patient. | P12. Workers at the Screening Centre are always willing to help you and the patient. |
|  | E13. Workers at the Screening Centre will never be too busy to respond to the patient’s or your requests. | P13. Workers at the Screening Centre are never too busy to respond to the patient’s or your requests. |
|  | **Assurance** (including competence, courtesy, credibility and security) | **Assurance** (including competence, courtesy, credibility and security) |
|  | E14. The behavior of the workers at the ICU will instill confidence in the patient and the family. | P14. The behavior of the workers at the ICU instills confidence in the patient and the family. |
|  | E15. You will feel safe for the patient’s care by the Screening Centre. | P15. You feel safe for the patient’s care by the Screening Centre. |
|  | E16. Workers at the Screening Centre will be consistently courteous with the patient and the family. | P16. Workers at the Screening Centre are consistently courteous with the patient and the family. |
|  | E17. Workers at the Screening Centre will have the knowledge to answer your questions. | P17. Workers at the Screening Centre have the knowledge to answer your questions. |
|  | **Empathy** (caring and individualized attention that the Screening centre provides to its patients and families) | **Empathy** (caring and individualized attention that the Screening centre provides to its patients and families) |
|  | E18. The Screening Centre will give you and the patient individual attention. | P18. The Screening Centre gives you and the patient individual attention. |
|  | E19. The Screening Centre will have operating hours convenient to its patients and families. | P19. The Screening Centre has operating hours convenient to its patients and families. |
|  | E20. The Screening Centre will have workers who give the patient and the family personal attention. | P20. The Screening Centre has workers who give the patient and the family personal attention. |
|  | E21. The Screening Centre will have the best interest of the patient and the family at heart. | P21. The Screening Centre has the best interest of the patient and the family at heart. |
|  | E22. Workers at the Screening Centre will understand the special needs of the patient and the family. | P22. Workers at the Screening Centre understand the special needs of the patient and the family. |
| Satisfaction | SAT1. I am satisfied with the screening service I received in this screening centre. | |
|  | SAT2. My decision to visit this screening centre has been a wise one. | |
|  | SAT3. The screening services I received corresponded to my current needs. | |
| Behavioral intention | BI1. I will recommend other people to use the screening services offered by this screening centre. | |
|  | BI2. I will consider this screening centre my first choice if I need medical services in the future. | |
|  | BI3. I will tell other people good things about screening centre. | |
| Discomfort | DIS13. The physician was [not] too rough when performing the screening. | |
|  | DIS14. I [did not have] a lot of pain during the procedure. | |
|  | DIS15. The procedure was more comfortable than I expected. | |
|  | DIS16. I was [not] embarrassed by the procedure. | |
| Baseline characteristics | BC1. Gender: 1. Male; 2. Female | |
|  | BC2. Age: | |
|  | BC3. Marital status: 1: Never married; 2: Married; 3: Separated (separated due to relationship discord); 4: Divorced; 5: Widowed | |
|  | BC4. Highest Educational Degree: 1: No formal schooling; 2: Primary school; 3: Junior high school; 4: Senior high school (including secondary/technical school); 5: College; 6: Undergraduate; 7: Postgraduate and above; 99: Not available | |
|  | BC5. Occupation: 1: Agricultural, forestry, livestock and fishery workers; 2: Workers; 3: Administrative and managerial staff; 4: Professional and technical staff (doctors, teachers, scientists); 5: Sales and service workers; 6: Domestic work; 7: Private owners; 8: Leaving/retired; 9: Non-working/lay-off; 10: Other or not easily classified; | |
|  | BC6. Residence: 1: Rural; 2: Urban | |
|  | BC7. Average annual family income (Yuan): 1: less than 10,000; 2: 1,000 ~ 29,000; 3: 3,000 ~ 49,000; 4: 5,000 ~ 69,000; 5: 7,000 ~ 89,000; 6: 9,000 ~ 109,000; 7: 11,000 ~ 199,000; 8: 200,000 and above | |
| Health-related characteristics | BC8. Health self-assessment：1.Very good; 2.Better; 3. General; 4. Poor | |
|  | BC9. I was very anxious about having the procedure: 1.Yes; 2. No | |
|  | BC10. Experience with endoscopy before: 1.Yes; 2. No |  |
|  | BC11. Time taken from home to our screening centre (minute): 1.Less than 30; 2. 30-60; 3. 60 and above | |
|  | BC12. Purpose of participating in the screening: 1.Medical check-ups without disease; 2. Disease review | |
|  | BC13. Experience of examination/visit at this Hospital before: 1.Yes; 2. No | |
|  | B14. Do you have any common chronic diseases (hypertension, diabetes or hyperlipidemia) ?: 1.Yes; 2. No | |

Except for the baseline characteristics, Each dimension and item has 1- 5 items and was scored by a 5-point Likert scale (from strongly agree to strongly disagree).

Table 4 Basic information on satisfaction survey respondents and non-respondents

| Variables |  | Total (%), *N*=627 |  | No-respondents (%), *N*=109 |  | Respondents (%), *N*=516 | *χ²* | *p-*value |
| --- | --- | --- | --- | --- | --- | --- | --- | --- |
| Gender |  |  |  |  |  |  | 1.855 | 0.173 |
| Male |  | 256(41.0) |  | 51(46.8) |  | 205(39.7) |  |  |
| Female |  | 369(59.0) |  | 58(53.2) |  | 311(60.3) |  |  |
| Age |  |  |  |  |  |  | 0.779 | 0.678 |
| 40~ |  | 108(17.3) |  | 22(20.4) |  | 86 (79.6) |  |  |
| 50~ |  | 309(49.4) |  | 52(16.8) |  | 257 (83.2) |  |  |
| 60~69 |  | 208(33.3) |  | 35(16.8) |  | 173 (83.2) |  |  |
| Marital Status |  |  |  |  |  |  | 0.616 | 0.432 |
| Currently married |  | 586(93.8) |  | 104(95.4) |  | 482 (93.4) |  |  |
| Others b |  | 39(6.2) |  | 5(4.6) |  | 34 (6.6) |  |  |
| Education level |  |  |  |  |  |  | 1.617 | 0.446 |
| Primary school and below |  | 236(37.8) |  | 47(43.1) |  | 189 (36.6) |  |  |
| Junior high school |  | 300(48.0) |  | 48(44.0) |  | 252 (48.8) |  |  |
| Senior high school and above |  | 89(14.2) |  | 14(12.8) |  | 75 (14.5) |  |  |
| Occupation Status |  |  |  |  |  |  | 0.161 | 0.688 |
| Unemployed c |  | 141(22.6) |  | 23(21.1) |  | 118 (22.9) |  |  |
| Employed d |  | 484(77.4) |  | 86(78.9) |  | 398 (77.1) |  |  |
| Residence |  |  |  |  |  |  | 6.086 | 0.014 |
| Rural |  | 524(83.8) |  | 100(91.7) |  | 424 (82.2) |  |  |
| Urban |  | 101(16.2) |  | 9(8.3) |  | 92 (17.8) |  |  |
| Family income (CNY/year) |  |  |  |  |  |  | 15.769 | 0.001 |
| <30000 |  | 70(11.2) |  | 6(5.5) |  | 64 (12.4) |  |  |
| 30000-69999 |  | 203(32.5) |  | 28(25.7) |  | 175 (33.9) |  |  |
| 70000-109999 |  | 183(29.3) |  | 48(44.0) |  | 135 (26.2) |  |  |
| ≥110000 |  | 169(27.0) |  | 27(24.8) |  | 142 (27.5) |  |  |
